# Acute Kidney Injury in Immune-Mediated Thrombotic Thrombocytopenic Purpura – Solving the Puzzle?

**DOI:** 10.1016/j.ekir.2025.11.018

**Published:** 2025-11-17

**Authors:** Juliane Schneider, Camille Schlesser, Michael Fischereder, Anke von Bergwelt-Baildon, Ulf Schönermarck

**Affiliations:** 1 Division of Nephrology, Department of Medicine IV, LMU University Hospital, LMU Munich, Munich, Germany

**To the Editor:**

Acute kidney injury (AKI) has been reported in ≤ 50% of acute episodes of immune thrombotic thrombocytopenic purpura (iTTP).^1^ Although the clinical course is usually mild and rapidly resolving, severe AKI with need for kidney replacement therapy can occur. In their study Robert *et al.*^2^ recently reported on 10 patients with the diagnosis of iTTP within a large cohort of 1157 patients with thrombotic microangiopathy (TMA) who underwent kidney biopsy. Histological signs of TMA were found in 7 patients (70%). In addition, other glomerular, vascular or tubulointerstitial lesions were seen in all but 1 biopsy. The conclusion that kidney involvement in iTTP is primarily secondary to renal TMA requires further considerations.

Patients with iTTP exhibit a higher prevalence of concomitant autoimmune diseases. For example, the association between systemic lupus erythematosus and iTTP is well-described; occurring in 6% to 7% of patients.^3^ In the present paper, 7 patients presented with concomitant autoimmune diseases (systemic lupus erythematosus, systemic sclerosis, vasculitis), infections, and/or malignant hypertension and kidney biopsies showed histological signs of proliferative glomerulonephritis or malignant hypertension in 5 patients.^2^ These disease entities are well-known trigger factors for complement activation and the development of atypical hemolytic uremic syndrome,^4^ explaining the occurrence of renal TMA and the development of severe AKI independent of the iTTP pathophysiology.

However, a possible link between ultralarge von Willebrand factor multimers, accumulating in the acute phase of iTTP, and the alternative complement pathway has recently been described, leading to complement activation and generation of terminal complement complexes (C5b-9).^5^ The presence of complement factor variants or risk haplotypes might further increase the risk for the development of atypical hemolytic uremic syndrome, but genetic testing was not performed.

In clinical practice, episodes of AKI in iTTP are usually mild and rapidly resolving, arguing against renal TMA as an underlying condition and favouring other factors such as prerenal causes, free hemoglobin toxicity, or acute tubular necrosis. Severe AKI, requirement of kidney replacement therapy, or prolonged kidney function impairment should prompt the search for other underlying diseases and rigorous performance of kidney biopsy. The suggested overlap between iTTP and other renal TMA forms make diagnosis and treatment decisions challenging.

## References

[1] Little D.J., Mathias L.M., Page E.E., Kremer Hovinga J.A., Vesely S.K., George J.N. (2017). Long-term kidney outcomes in patients with acquired thrombotic thrombocytopenic purpura. Kidney Int Rep.

[2] Robert M., Maisons V., Rabant M. (2025). New insights into renal involvement during immune-mediated thrombotic thrombocytopenic purpura. Kidney Int Rep.

[3] Zhang K., Zhao P., Huang B. (2025). Clinical characteristics and treatment response of patients with SLE complicated with thrombotic thrombocytopenic purpura. Lupus Sci Med.

[4] Kang H.G., Hsu D., Kato N. (2025). Management of atypical haemolytic uraemic syndrome with triggers: diagnostic and treatment algorithms from an Asia-pacific perspective. Nephrology (Carlton).

[5] Turner N., Nolasco L., Nolasco J., Sartain S., Moake J. (2014). Thrombotic microangiopathies and the linkage between von Willebrand factor and the alternative complement pathway. Semin Thromb Hemost.

